# Mobile health application reduces dialysis catheter service time for patients with newly created arteriovenous fistulas

**DOI:** 10.3389/fmed.2026.1746883

**Published:** 2026-03-24

**Authors:** Liuyin Chen, Yuanchuan Hu, Yu Zhang, Fang Liu, Qining Fu, Jun Shen

**Affiliations:** 1 Department of Nursing, First Affiliated Hospital of Chongqing Medical University, Chongqing, China; 2 Department of Vascular Surgery, First Affiliated Hospital of Chongqing Medical University, Chongqing, China

**Keywords:** arteriovenous fistula, follow-up management, intelligent nursing care, mobile health application, vascular access

## Abstract

**Background and hypothesis:**

This study aimed to explore the effect of a mobile health application in the management of patients with newly created arteriovenous fistulas (AVFs).

**Methods:**

Hemodialysis patients who started dialysis with a dialysis catheter and received AVF creation at our hospital between August 2023 and April 2024, with follow-up via a mobile health application, were selected as the study group, while patients who received follow-up through traditional phone calls from January 2023 to July 2023 were selected as the control group. A 1:1 propensity score matching (PSM) analysis was conducted. The time from AVF creation to dialysis catheter removal [central venous catheter (CVC) days], the time to first AVF cannulation, and complications within 6 months postoperatively were compared between the two groups.

**Results:**

In the PSM cohort, the CVC days were 61.5 (53.5, 68.8) in the mobile health application group and 93.0 (75.5, 100.0) in the control group (*p* < 0.05). The time to first cannulation was 57.0 (49.0, 62.8) days in the mobile health application group and 83.0 (69.3, 92.0) days in the control group. There was no significant difference in complications between the two groups within 6 months. These results were independent of patients’ age, education level, and dialysis hospital level.

**Conclusion:**

Mobile health applications can help reduce the number of CVC days and promote earlier cannulation of newly created AVFs without increasing the risk of complications.

## Graphical abstract

**Figure:**
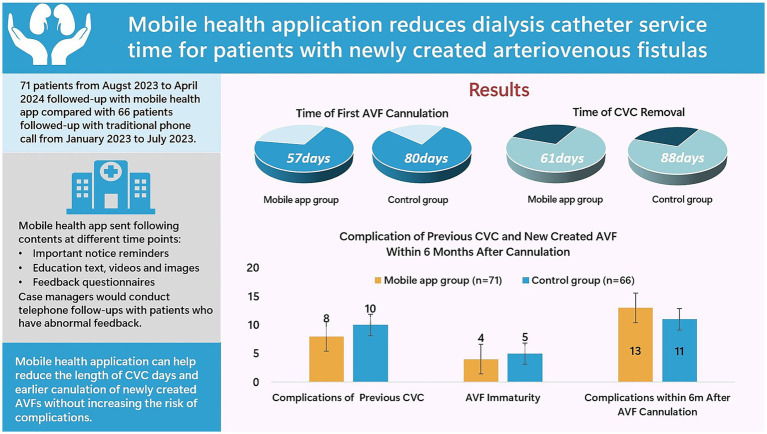

## 1 Introduction

According to the latest figures released, by the end of 2024, the total number of hemodialysis patients in China had surpassed 1,027,000, with an additional 220,000 new cases reported in 2024 alone. As China’s aging population continues to grow, it is anticipated that the number of dialysis patients will further increase. Hemodialysis is the primary treatment for end-stage renal disease (ESRD), while vascular access is often referred to as the “lifeline” for maintenance hemodialysis (MHD) patients (1–4). Arteriovenous fistula (AVF), the most commonly used vascular access for MHD patients (5, 6), typically requires a maturation period before cannulation after creation. Consequently, patients who have already initiated dialysis frequently rely on central venous catheters (CVCs) as a temporary measure. Various guidelines emphasize the importance of removing temporary catheters as soon as a newly created AVF can be cannulated, due to the long-term negative impact of catheter complications on prognosis. However, delays in the timely removal of temporary catheters remain a common issue. Mobile health applications have emerged as a novel approach to follow-up management, and our center has been using them for the follow-up of hemodialysis patients with vascular access in recent years.

This study evaluated the mobile health application implemented at our hospital for MHD patients with newly created AVFs who had already initiated dialysis using CVCs. The aim was to investigate its effects on the CVC service time (CVC days), AVF initial cannulation time, and early AVF-related complications within the first 6 months. The findings are presented in the latter sections.

## 2 Materials and methods

### 2.1 Study setting

In this retrospective study, we included all patients who had already initiated dialysis with a CVC and subsequently underwent new AVF creation at our hospital between August 2023 and April 2024, following the introduction of the mobile health application. For comparison, patients who underwent similar procedures between January 2023 and July 2023, prior to the introduction of the mobile health application, were retrospectively enrolled as the control group.

The inclusion criteria were as follows: (1) patients who had initiated dialysis with a CVC and subsequently underwent new AVF creation at our center, (2) individuals aged 18 years or older, (3) patients or their family members who possessed smartphones, and (4) patients with completely intact cognitive and behavioral capabilities. The exclusion criteria were as follows: (1) patients or their families without smartphones, (2) individuals diagnosed with mental disorders or impairments in cognitive and expressive functions, (3) patients or their families unable to proficiently use the mobile health application, and (4) patients unwilling to participate in follow-up through the mobile health application. Ethical approval for this study was obtained from the Institutional Ethics Committee of the First Affiliated Hospital of Chongqing Medical University (Project Identification Code: 2023-403). All studies were carried out in accordance with the relevant provisions of the Declaration of Helsinki. Participants were fully informed about the study, including details regarding the confidentiality of their information. They were also advised of their right to withdraw from the study at any time.

Patient’s demographic data, including age, sex, education level, residence location, and dialysis facility, were collected. Propensity score matching (PSM) was performed using the group of the patient as the dependent variable and demographic characteristics as the independent variables. The caliper value was 0.05 times the standard deviation of the logit-transformed propensity score, with a matching ratio of 1:1. This ensured that the standardized mean difference (SMD) of confounding factors between the two groups after matching was less than 0.1.

### 2.2 Postoperative education and follow-up methods

Prior to the launch of the mobile health application in August 2023, the management protocol for patients with newly created AVF was as follows: Upon discharge, nurses provided both oral and paper discharge instructions, including guidance on incision management, limb functional exercises, precautions, daily care for the limb with the AVF, and methods of self-monitoring the AVF. Information regarding approximate AVF maturation time, the risks associated with long-term indwelling catheters, and common complications of AVF was also provided. In accordance with hospital protocols, telephone follow-ups were conducted within 7 days post-discharge according to established follow-up procedures. Subsequent telephone follow-ups were performed every 3 months after surgery.

After August 2023, our center initiated a mobile health application for patient follow-up. Upon patient discharge, the case manager created a cloud-based file within the system and selected the “New AVF with dialysis catheter” follow-up and patient education plan. Based on predefined settings, the mobile health application disseminates follow-up text messages and educational content to the patients or their caregivers. The educational materials include text, videos, and images. (1) On the first day post-discharge, the application provides postoperative information, including postoperative precautions and AVF self-monitoring methods. On the third day post-discharge, the application delivers essential knowledge about AVF self-care, as along with reminders for wound dressing changes. On the fifth day, a video demonstrating hand exercises to promote AVF maturation is sent out to patients. Patients are asked to provide feedback regarding fistula thrill and wound condition; if any abnormalities are detected, the case manager contacts the patient directly. (2) A reminder concerning suture removal is issued on postoperative day (POD) 14, on POD 21, patients are asked to report on fistula thrill and wound status. (3) On POD 42, information regarding the initiation of AVF cannulation is provided, including the evaluation criteria for AVF maturation, the significance of timely CVC removal, and precautions following AVF cannulation. Patients are asked to provide feedback regarding fistula thrill or any other abnormal condition. Case managers assess whether further medical intervention is warranted. (4) On POD 60, patients are asked to report whether they have initiated use of their AVFs, along with current functional parameters, such as blood flow, venous pressure, and hemostasis time at needle puncture sites. For those still reliant on catheters, case managers conduct telephonic follow-ups to clarify underlying reasons and coordinate with medical staff at patients’ dialysis centers or arrange potential hospital visits (5). Subsequent Follow-Up: Thereafter, each patient’s AVF functional status is evaluated every month using the mobile health application, along with monitoring for any abnormalities. Educational materials from the patient education content library are also provided. Every 6 months, the mobile health application sends reminders for hospital-based surveillance.

### 2.3 Data collection and definitions

The primary aim of the study was to compare the two groups in terms of CVC days and the time to initial AVF cannulation. Data on suture removal, complications related to previous CVC and newly created AVF within 6 months after cannulation initiation, and corresponding interventions were also collected. Complications were primarily based on self-reports of patients through the two follow-up methods. Cases requiring further surgical intervention were obtained from the electronic medical record system.

CVC Days: Given the variability in the actual timing of catheter implantation prior to AVF creation among different patients, the CVC days in this study were solely calculated from the day of AVF creation to the day of CVC actual removal.

The time of AVF initial cannulation: This refers to the interval from the day of AVF creation until its first cannulation, irrespective of whether the dialysis was successfully completed.

### 2.4 Statistical analysis

Statistical analyses were conducted using SPSS version 26. Quantitative data that followed a normal distribution were presented as mean ± standard deviation (M ± SD), while non-normally distributed data were reported as median (interquartile range, IQR). For intergroup comparisons, normally distributed data were analyzed using independent samples t-tests, whereas non-normally distributed data were compared using the Mann–Whitney U-test. Categorical data were summarized as frequency and percentage (%), with intergroup comparisons performed using the chi-square (χ^2^) test. A multivariate logistic regression analysis was used to determine the influence of variates on the primary endpoint event. For complications, a *post-hoc* power analysis was conducted using the two-tailed chi-square test, with the significance level α set at 0.05. A *p*-value of less than 0.05 was considered statistically significant.

## 3 Results

### 3.1 Demography

There were 94 patients receiving new AVF creation, and 76 (80.9%) of them participated in follow-ups through mobile health applications from August 2023 to April 2024. Excluding 5 patients who did not use CVC, 71 patients were included in the mobile application group.

There were 72 patients receiving new AVF creation from January 2023 to July 2023. Excluding 2 patients who did not use CVC and 4 patients lost to follow-up, 66 patients were included in the control group.

PSM based on baseline data, such as age, gender, education level, residential location, level of dialysis hospital, and public or private status, was used to separate the PSM cohort from the main cohort. After 1:1 matching, each group had 54 patients remaining, totaling 108 patients.

**Figure 1:**
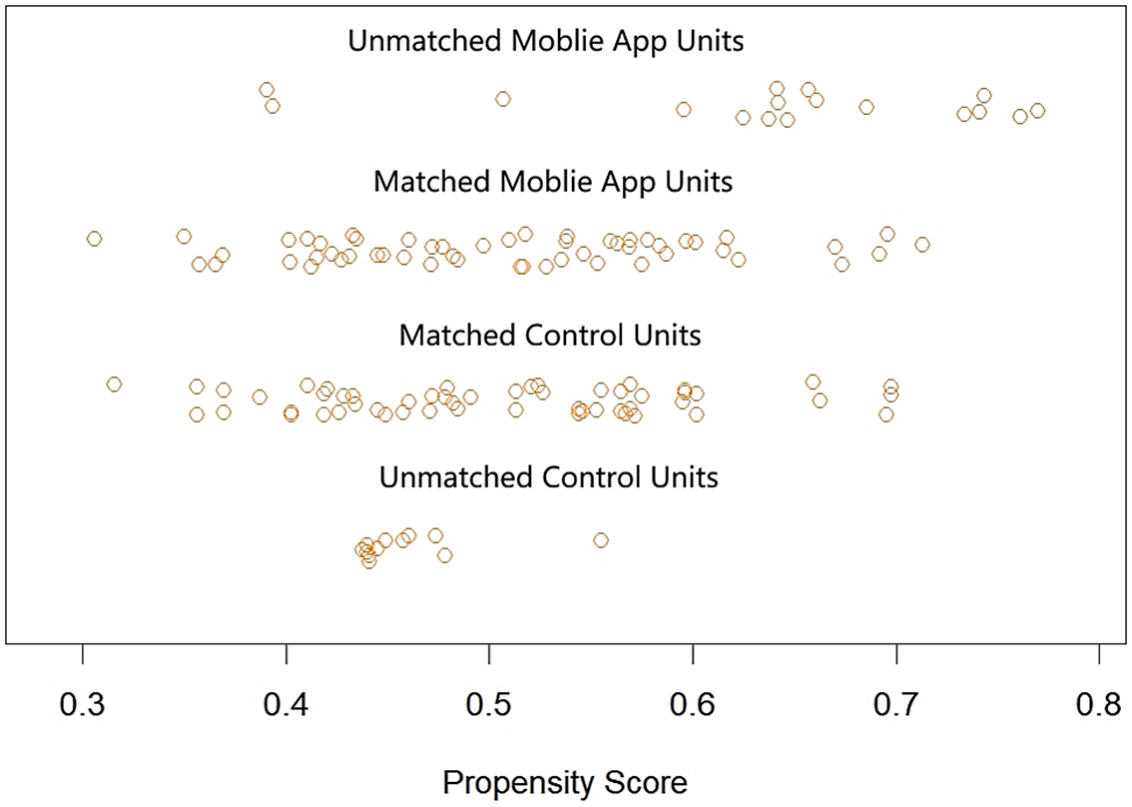
Distribution of propensity scores.

Distribution of propensity scores is shown in Figure 1, and the demography of the PSM cohort is displayed in Table 1. There were no statistically significant differences in all baseline variables (*p* > 0.05), and the baseline characteristics were balanced between the groups (*p* > 0.05).

**Table 1:** Comparison of baseline characteristics between the two groups before and after propensity score matching (PSM).

Patient Characteristics			Before PSM				After PSM		
		Mobile application group (*n* = 71)	Control group (*n* = 66)	*Z*/χ^2^ value	*P*	Mobile application group (*n* = 54)	Control group (*n* = 54)	*Z*/χ^2^ value	*P*
Age (in years)		58.0 (49.0, 70.0)	64.5 (55.3, 70.3)	1.85	0.06	60.00 (54.25, 71.00)	63.50 (53.00, 71.75)	0.33	0.75
Sex	Female	32 (45.07)	31 (46.97)	0.05	0.82	25 (46.30)	27 (50.00)	0.15	0.70
Level of education	Illiteracy	17 (23.944)	19 (28.788)	7.27	0.20	28 (51.85)	28 (51.85)	0.00	1.00
	Primary school	24 (33.803)	24 (36.364)			26 (48.15)	26 (48.15)		
	Junior high school	16 (22.535)	16 (24.242)			16 (29.63)	16 (29.63)		
	Senior high school	8 (11.268)	4 (6.061)			21 (38.89)	17 (31.48)		
	Vocational college	1 (1.408)	3 (4.545)			10 (18.52)	14 (25.93)		
	Collegiate	5 (7.042)	0 (0.000)			5 (9.26)	4 (7.41)		
Residential location	Village	35 (49.296)	36 (54.545)	0.37	0.54	1 (1.85)	3 (5.56)	-	0.73
	City	36 (50.704)	30 (45.455)			1 (1.85)	0 (0.00)		
Dialysis center level	Public tertiary hospital	27 (38.028)	26 (39.394)	0.5	0.78	17 (31.48)	21 (38.89)	0.69	0.71
	Public secondary hospital	22 (30.986)	17 (25.758)			17 (31.48)	16 (29.63)		
	Private hospital	22 (30.986)	23 (34.848)			20 (37.04)	17 (31.48)		

Z, Mann–Whitney test; χ^2^, chi-square test; −, Fisher exact.

M, median; Q_1_, first Quartile; Q_3_, third quartile.

#### 3.1.1 Mobile health application reduced CVC days and advanced time of AVF initial cannulation

The CVC days for the mobile application group were 61.0 days (IQR: 50.0, 67.0), while those of the control group were 88.0 days (IQR: 75.0, 99.3). In the PSM cohort, the CVC days for the mobile app group were 61.5 days (IQR: 53.5, 68.8), which was significantly lower than that of the control group (93.0 days, IQR: 75.5, 100.0).

**Figure 2:**
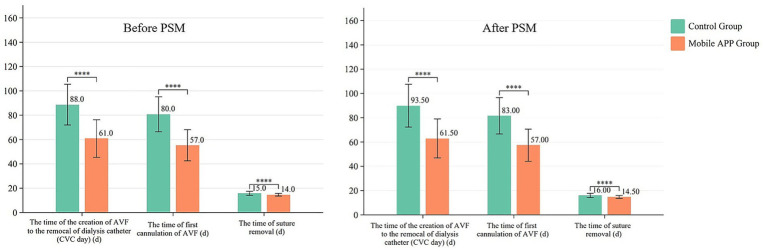
Comparison of CVC days, AVF initial cannulation time, and wound suture removal time between the two groups before and after propensity score matching (PSM). Continuous variables are presented as median (interquartile range, IQR: P25, P75), and * denotes statistically significant results. AVF, arteriovenous fistula; CVC, central venous catheter.

The time of AVF initial cannulation was 57.0 days (IQR: 45.0, 62.0) post-operation in the mobile application group and 80.0 days (IQR: 69.0, 92.0) in the control group. In the PSM cohort, it was 57.0 days (IQR: 49.0, 62.8) post-operation in the mobile application group, which was significantly shorter compared to that of the control group (83.0, IQR: 69.3, 92.00) (Figure 2).

The suture removal time was 14.0 days (IQR: 14.0, 15.0) for the mobile application group and 15.0 days (IQR: 15.0, 16.3) for the control group. In the PSM cohort, it was 14.5 days (IQR: 14.0, 15.0) in the mobile application group and 16.0 days (IQR: 15.0, 17.0) in the control group (IQR: 69.25, 92.00). There was no statistically significant difference between the two groups in terms of clinical outcomes.

A multivariate logistic regression analysis showed consistent results: the mobile health application was associated with shorter CVC days and earlier AVF initial cannulation. Education level may have had a certain degree of influence on the outcomes; however, only patients with “vocational college” education showed a significant difference in CVC days. This result cannot be interpreted as lower educational levels are more likely to affect the outcome.

#### 3.1.2 Access complications of previous CVC and newly created AVF within the first 6 months

There were 8 and 10 dialysis catheter-related complications in the mobile application group and control group, respectively. Catheter dysfunction was the predominant complication, occurring in 6 cases in the mobile application group and 8 cases in the control group. Of these, 4 cases continued using the catheter after thrombolytic therapy with urokinase, while 2 cases required catheter replacement. Additionally, 8 cases had their CVC removed and were transitioned to AVF as vascular access. Catheter dislodgment occurred in 3 cases (1 in the mobile application group and 2 in the control group), with 1 case receiving catheter replacement at a different site and 2 cases transferred to AVF as access. One case of catheter infection was observed in the mobile application group, which was managed with antibiotic lock and oral antibiotics and transferred to AVF 1 week later.

A total of 9 patients were diagnosed as “immaturity” and were postponed cannulation in local hospitals, comprising 4 cases in the mobile application group and 5 cases in the control group. Each group included four patients with “insufficient blood flow,” observed at an average duration of 53.75 days post-operation. None of these patients received any additional interventions and were only encouraged to continue physical exercise. Ultimately, all these patients initiated use of the AVF at an average of 85.75 days after surgery. One patient was evaluated by a local hospital as having a “deep vein” AVF 28 days after surgery. The patient did not undergo further surgical intervention and finally received the first AVF cannulation 40 days post-operation.

Within the first 6 months of AVF cannulation, a total of 24 complications were reported, with 13 cases in the mobile application group and 11 cases in the control group. The most common complication was flow dysfunction (seven cases in the mobile application group and eight cases in the control group). Additionally, thrombosis developed in six cases (three from each group). All the above were successfully managed through endovascular intervention. One patient in each group experienced limb swelling where the AVF was located; both patients had previous CVCs placed on the ipsilateral side of their created AVF. Central venous stenosis was diagnosed by angiography, and swelling subsided after endovascular angioplasty. Furthermore, four patients developed localized infiltrations post-cannulation. All of them were in the mobile application group and were gradually resolved following local physical therapy and did not impact subsequent AVF cannulation.

All these complications, complications per patient, and interventions for complications per patient (Table 2) revealed no statistically significant difference between the two groups (*p* > 0.05). There was still no statistically significant difference in the PSM cohort.

**Table 2:** Complications of previous CVC and newly created AVF within 6 months after cannulation between the two groups.

Complication		Mobile application group (*n* = 71)	Control group (*n* = 66)	χ^2^ value	*p*
Complications of previous CVC		8 (11.268)	10 (15.152)	0.452	0.501
	Catheter dysfunction	6 (8.451)	8 (12.121)	1.851	0.604
	Catheter infection	1 (1.408)	0 (0.000)		
	Catheter dislodgment	1 (1.408)	2 (3.030)		
AVF immaturity		4 (5.634)	5 (7.576)	0.210	0.647
	Deep vein	0 (0.00)	1 (1.515)	1.100	0.577
	Flow dysfunction	4 (5.634)	4 (6.061)		
Complications within 6 months after AVF cannulation		13 (18.310)	11 (16.667)	0.064	0.800
	Thrombosis	3 (4.225)	3 (4.545)	0.008	0.927
	Flow dysfunction	7 (9.859)	8 (12.121)	0.180	0.672
	CVS	1 (1.408)	1 (1.515)	0.003	0.959
	Infiltration hematoma	4 (5.634)	0 (0.000)	3.830	0.050
Complications per patient		0.240 ± 0.550	0.230 ± 0.550	0.130	0.897
Interventions for complication		10 (14.085)	11 (16.667)	2.949	0.229
Interventions for complications per patient		1.000 ± 0.710	1.360 ± 0.500	−1.424	0.168

AVF, arteriovenous fistula; CVC, central venous catheter; CVS, central venous stenosis.

The complication incidence rates were 11.27% in the mobile application group and 15.15% in the control group. The calculated test power was 0.103. The rate difference of complications between the two groups was 0.0388, with a 95% confidence interval of −0.075 to 0.152. The difference was not statistically significant.

## 4 Discussion

For patients initiating hemodialysis with CVC, the optimal choice of vascular access involves balancing shorter CVC service duration with higher long-term patency, fewer interventions, and easier vascular access cannulation (7–9).

Due to the insidious onset of chronic kidney disease (CKD), a significant proportion of patients are already requiring immediate kidney replacement therapy at their initial diagnosis. In China, hemodialysis is the most prevalent form of renal replacement therapy, while CVCs usually serve as the primary vascular access for these patients. As guidelines suggest, the majority of CVCs are only a temporary transitional solution, and patients should be subsequently transferred to AVFs or arteriovenous grafts. Transitional CVCs are also needed in scenarios where patients have new AVFs created as the previous AVFs are abandoned.

Given the focus on complications associated with prolonged CVC retention, particularly the risk of central venous lesions, many guidelines recommend early removal of such transitional CVCs. In our series, two patients developed central venous stenosis within a short period following AVF creation, which was highly likely due to previous CVC use. This further underscores the critical importance of timely CVC removal.

However, prolonged catheter service is a prevalent issue. As our study showed, a number of patients started their AVF cannulation only after experiencing catheter complications. Additionally, given that no significant differences were observed between the mobile application group and the control group regarding age, education level, and dialysis center classification (public or private and the level of hospital), it can be deduced that these factors did not significantly influence the attention given to transitional CVC removal. We observed cases in which catheters were in service 1–2 years post-AVF creation until catheter failure occurred.

To address this issue, on the one hand, it is necessary to further train and elevate the professional level of medical staff; on the other hand, patient education should be intensified to encourage more participation in self-management of vascular access (10, 11), thereby collectively increasing awareness regarding timely CVC removal and early AVF cannulation. Nevertheless, due to the entrenched influence of traditional medical practices and concepts, further efforts are needed to achieve this transformation. This study reveals that, regardless of the mobile application group or the control group, there was no significant difference in the time of suture removal. This reflects that there is a consensus on suture removal among medical staff, but a lack of consensus remains regarding the timing of initial AVF cannulation and CVC removal.

Even guidelines from different countries exhibit significant divergence regarding initial AVF cannulation time. The European Society for Vascular Surgery guideline recommends conducting appropriate clinical examinations of the AVF 4–6 weeks after creation, prior to initial cannulation. Cannulation may be considered between 2 and 4 weeks post-surgery in certain cases with close monitoring (12). The expert consensus on vascular access for hemodialysis in China (second edition) recommends 8–12 weeks post-AVF creation. If plastic cannulae are used, this timeline can be shortened to 2–3 weeks post-surgery (13). Conversely, the Japanese Society for Dialysis Therapy Guidelines suggest that cannulation can be performed as early as 2 weeks after AVF creation (14), although early cannulation carries a higher risk of cannulation complications. In our study, more cases of infiltration were observed in the mobile application group (as these were based on patients’ self-reports, the severity grading was unknown), nonetheless, this did not show statistical significance between the two groups. These variations among guidelines, along with concerns regarding early cannulation complications, may lead medical staff to postpone AVF cannulation in clinical practice. In our study, the majority of patients were initially diagnosed with “immaturity” by local hospitals. However, none of these patients received any special intervention and eventually had their AVF normally cannulated after a period of time. It is likely that a considerable proportion of the AVFs labeled as “immaturity” were classified based on subjective judgment by medical staff rather than objective inspection, in order to postpone cannulation and increase the safety and success rate of the first cannulation. This also indicates that more efforts are needed to raise awareness of timely CVC removal and to practice the “ESKD LIFE PLAN” advocated by the KDOQI clinical practice guideline for vascular access 2019 update (15). Traditional follow-up is limited by time constraints and understaffing, particularly as the number of patients increases, and they come from multiple hospitals and dialysis centers. Therefore, there is an urgent need to introduce new methods to address this situation.

The introduction of mobile health applications in follow-up and patient education represents an innovative approach to patient care. This approach effectively addresses the shortage of medical staff for out-of-hospital management of chronic disease patients. Mobile health applications are not constrained by time or location, thereby providing continuous and intelligent services, and are experiencing rapid growth in chronic disease management (16, 17). Research focusing on the management of patients with chronic kidney disease has shown that mobile health applications improve patients’ self-management scores, reduce the mean systolic blood pressure, and increase patient satisfaction (18).

Our study revealed that the mobile health application facilitated earlier AVF cannulation and reduced CVC service time without increasing the risk of AVF complications within the first 6 months of use. This underscores the significant value of mobile health applications in patient care. The mobile health application provided continuous care, timely and precise nursing guidance, and regular follow-ups. It enhanced the interaction between patients and medical staff, which is conducive to the timely discovery and treatment of existing problems of vascular access and potentially improves dialysis quality and patients’ quality of life (19–21). Moreover, rich and diverse educational content delivered through the application helps patients develop a correct understanding of hemodialysis access, promotes active participation in self-management, and facilitates adherence to subsequent monitoring and follow-up, thereby maximizing patient benefits.

In our study, the AVF initial cannulation time in the mobile application group was 57.0 days. Given that the time for AVF maturation is highly individualized, there may be opportunities to further reduce the duration for certain patients. The guideline of the Japanese Society for Dialysis Therapy recommends that cannulation can be performed 2 weeks after AVF creation (14). Plastic cannulae could play a helpful role in earlier AVF cannulation (22). A study by Buzzell M showed that early postoperative follow-up for the evaluation of AVF maturation is associated with reduced time to first successful cannulation of AVF and reduced time to catheter-free dialysis. Further research is warranted to explore how mobile health applications can support personalized assessment for AVF maturation and optimize first cannulation.

This study still has the following limitations. First, the inclusion criteria related to smartphone ownership and application use may introduce selection bias. Since the research premise requires that patients be willing to use mobile applications, the inclusion criteria automatically selected patients who were more concerned about their own health and more compliant with medical advice. Digital literacy of patients could also affect outcomes. Second, the intervention and control groups were derived from different time periods, introducing potential bias from secular trends and changes in clinical practice that cannot be fully controlled in the current design. Third, due to the limitations of clinical practical conditions, the sample size was small. Considering the low occurrence rate of complications resulting in a relatively low test efficacy, the results should be interpreted with caution. Further validation is needed through large-sample, multicenter studies.

In the future, there is considerable potential for mobile health applications in the early detection of vascular access complications and the reduction of the risk of severe complications such as infections and thrombosis. The integration of artificial intelligence has the potential to transform current practices in hemodialysis vascular access management, providing greater benefits for MHD patients.

## Data Availability

The raw data supporting the conclusions of this article are available from the corresponding author upon reasonable request.
